# Effects of Cr and Zr Addition on Microstructures, Compressive Properties, and Abrasive Wear Behaviors of In Situ TiB_2_/Cu Cermets

**DOI:** 10.3390/ma11081464

**Published:** 2018-08-17

**Authors:** Feng Qiu, Xiangzheng Duan, Baixin Dong, Hongyu Yang, Jianbang Lu, Xiujuan Li

**Affiliations:** 1 Key Laboratory of Automobile Materials, Ministry of Education and Department of Materials Science and Engineering, Jilin University, Changchun 130025, China; qiufeng@jlu.edu.cn (F.Q.); duanxz17@mails.jlu.edu.cn (X.D.); dongbx1614@mails.jlu.edu.cn (B.D.); yanghy@just.edu.cn (H.Y.); boshilujianbang@163.com (J.L.); 2 Key Laboratory of Bionic Engineering, Ministry of Education, Jilin University, Changchun 130025, China; 3 School of Materials Science and Engineering, Jiangsu University of Science and Technology, Jiangsu 212003, China; 4 Welding Division of Manufacture Engineering Department, Automotive Engineering Corporation, Tianjin 300113, China; 5 Qingdao Automotive Research Institute, Jilin University, Qingdao 266000, China

**Keywords:** combustion, in situ, cermets, compression property, abrasive wear

## Abstract

In situ micro-TiB_2_/Cu cermets with a different TiB_2_ content (40, 50, and 60 vol %) were successfully fabricated by combustion synthesis (CS) and hot press consolidation in Cu-Ti-B systems. In addition, different contents of Cr and Zr were added to the Cu-Ti-B systems. The microstructure, mechanical properties, and abrasive wear properties of the TiB_2_/Cu cermets were investigated. As the ceramic content increased, the yield strength and compressive strength of the cermets were found to increase, while the strain decreased. An increase in load and abrasive particle size caused the wear volume loss of the TiB_2_/Cu cermets to increase. When the ceramic content was 60 vol %, the wear resistance of the TiB_2_/Cu cermets was 3.3 times higher than that of pure copper. The addition of the alloying elements Zr and Cr had a significant effect on the mechanical properties of the cermets. When the Cr content was 5 wt %, the yield strength, ultimate compressive strength, and microhardness of the cermets reached a maximum of 997 MPa, 1183 MPa, and 491 Hv, respectively. Correspondingly, when the Zr content was 5 wt %, those three values reached 1764 MPa, 1967 MPa, and 655 Hv, respectively, which are 871 MPa, 919 MPa, and 223 Hv higher than those of the unalloyed cermets. The wear mechanism of the in-situ TiB_2_/Cu cermets, and the mechanisms by which the strength and wear resistance were enhanced by the addition of Zr, were preliminarily revealed.

## 1. Introduction

With the development of large-scale integrated circuits and high-power precision instruments, the application of new electronic packaging and heat sink materials has received widespread attention [1,2,3,4]. First and foremost, an electronic packaging and heat sink material must have a coefficient of thermal expansion matching the semiconductor or chip material, and better thermal conductivity [5,6]. In addition, it must have good airtightness, high mechanical strength, and good chemical stability. Due to its conductivity and ductility, Cu is widely used in electronic packaging and heat sink materials [7]. However, due to its low hardness and strength, poor wear resistance, easy oxidation, and easy softening and deformation above 500 °C, Cu tends to be rapidly consumed, which limits its widespread application [8,9,10]. To avoid the above drawbacks, ceramic particles are usually added to the copper matrix [11]. Ceramic particle-reinforced Cu matrix cermets have high strength and hardness, good wear resistance, and oxidation resistance, a low coefficient of thermal expansion, and a simple preparation process, spurring their rapid development in recent years [12]. TiB_2_ ceramic particles have a high melting point, high hardness, high conductivity, high modulus, low density, and good chemical stability [13,14,15,16]. Therefore, adding TiB_2_ particles as a reinforcing phase into the Cu matrix not only maintains the good thermal and electrical conductivity of Cu [17,18], but also improves the strength and wear resistance of the material [19,20,21,22]. The application prospects of TiB_2_ are very broad. The traditional methods of preparing TiB_2_ ceramics are sintering, mechanical alloying, molten salt-assisted synthesis, and combustion synthesis (CS) [23,24,25]. CS has been extensively researched due to its simplicity, low energy usage, high production efficiency, and high product purity [26,27]. However, cermets prepared by the CS method are highly porous, and the distribution of micron-scale ceramics in the matrix is not uniform [28,29,30,31]. At the same time as the in situ reaction, the axial compression is beneficial to the densification of composites. For instance, Liang et al. [18,32] studied the in situ mechanism of TiC*_x_* in a Cu-Ti-C system and successfully prepared TiC*_x_*-reinforced Cu matrix composites by the combustion synthesis and hot press methods. Liang et al. [20] investigated the thermal explosion reaction behavior of Cu-Ti-C systems with different Ti and C particle sizes, and the found that the sizes of C particles have a great influence on the ignition temperatures of the system. Zhang et al. [8] studied that the compression properties and electrical conductivity of in situ 20 vol % nano-sized TiC*_x_*/Cu composites fabricated via combustion synthesis and hot press in Cu-Ti-CNTs system at various particles size and morphology. In our previous works [33], 40–60 vol % TiC-TiB_2_ with an average particle size of 1.5 µm are introduced into molten Cu by combustion synthesis and hot press method. Compacted reinforced TiC-TiB_2_/Cu cermets composites with homogeneous dispersion have been obtained. Zhang et al. [34] revealed that the trace elements Cr and Zr are the best strengthening elements of Cu alloys. In this study, a hot pressing-sintering in-situ reaction is used to prevent formation of voids in cermets [35,36]. High energy ball milling is used to activate boron, which is beneficial to reducing the reaction temperature of Cu-Ti-B systems [37,38,39], reducing the size of ceramic particles, and promoting the dispersion of the reinforcement phase in the matrix [40,41]. The elements Zr and Cr can be added to Cu-Ti-B systems to refine the ceramic particles and to promote the interfacial bonding between TiB_2_ ceramic particles and the Cu matrix [42,43,44,45]. However, the synthesis of cermets by the CS method is hindered by the difficulty of pressurization and densification [46,47,48,49]. At present, there are few reports of the synthesis of in situ TiB_2_/Cu cermets by CS, combined with hot press consolidation. 

In this paper, the Cu-Ti-B system was selected as the research object, and in situ TiB_2_/Cu cermets with high hardness, strength, and wear resistance were successfully fabricated by CS combined with hot press consolidation. The effects of TiB_2_ ceramic particle content on the microstructure, compressive properties, and abrasive wear properties of the cermets were investigated. The effect of different Cr and Zr contents on the microstructures, compression properties, and abrasive wear behavior of the cermets is also discussed. The wear mechanism of the in-situ TiB_2_/Cu cermets, and the mechanisms by which their strength and wear resistance were greatly enhanced by the addition of Zr, were preliminarily revealed. 

## 2. Experimental Procedures

**Figure 1:**
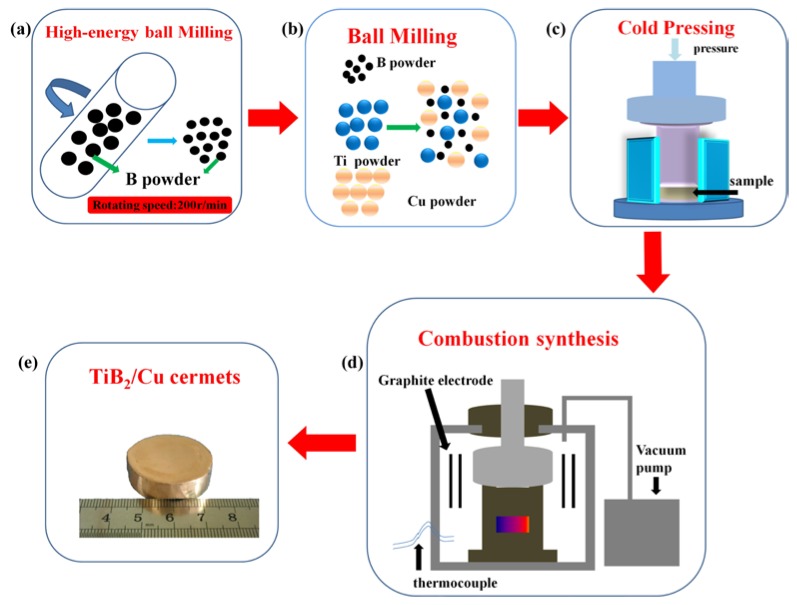
Schematic diagram of the preparation principle of the TiB_2_/Cu cermets. (**a**) High energy ball milling of B powder; (**b**) ball milling treatment of mixed powder; (**c**) cold pressing of mixed powder into a cylinder; (**d**) preparation of micro-sized TiB_2_/Cu cermets by hot press sintering of the compacts; (**e**) sample of TiB_2_/Cu cermets.

Figure 1 is a schematic diagram of the principle of preparation of TiB_2_/Cu cermets. The raw materials used were commercial copper powder (99.7 wt % purity, ~47 μm in diameter), titanium powder (99.5 wt % purity, ~25 μm in diameter), boron (B) powder (98 wt % purity, ~1 μm in diameter), Zr powder (99.5 wt % purity, ~47 μm in diameter), and Cr powder (99.7 wt % purity, ~47 μm in diameter). As shown in Figure 1a, high energy ball milling (Planetary ball mill, weight of balls to powder ratio was 50:1, zirconia grinding ball, Gosta, Siping, China) was used to activate the boron powder; the rotating speed was 200 rpm over 120 min. The Cr and Zr powders at weight percentages of 3, 5, or 7 wt % were added to the mixed powders containing ceramic particles at 50 vol %, and the mixtures were thoroughly homogenized by the method shown in Figure 1b. The materials were mixed sufficiently with low-speed ball milling (24 h, approximately 50 rpm, planetary ball mill, weight of balls to powder ratio was 10:1, zirconia grinding ball) and cold pressed into cylindrical samples afterwards. Figure 1c shows the cold press process, in which each mixture was wrapped with tin foil and pressed into a cylinder (Φ = 28 mm × 35 mm) on a cold press with pressure of about 75 MPa. Then, the sample was placed in a vacuum thermal explosion furnace, as shown in Figure 1d, and heated at a rate of 30 K/min under vacuum conditions. When the barometric pressure showed a sharp increase, it indicated that the sample had reacted. The pressure of about 40 MPa was immediately applied to the sample for 60 s. Finally, the TiB_2_/Cu cermets were successfully obtained by cooling to room temperature. 

The phase constituents of the products were investigated by X-ray diffraction (XRD, Model D/Max 2500PC, Rigaku, Tokyo, Japan) with Cu Kα radiation, and their microstructure was investigated by scanning electron microscopy (SEM, Model Evo18, Carl Zeiss, Oberkochen, Germany). A servo-hydraulic materials testing system (Model MTS 810, MTS, Minneapolis, MN, USA) was used to perform the compression test with a strain rate of 1 × 10^−4^ s^−1^ at room temperature. Cylindrical specimens with a diameter of 3 mm and a height of 6 mm were used for the compression tests. A Vickers hardness tester (HVS-1000A, Huayin, Laizhou, China) was used to test the microhardness of the TiB_2_/Cu cermets with the load of 7 N for 10 s, and each sample was tested for 10 times.

**Figure 2:**
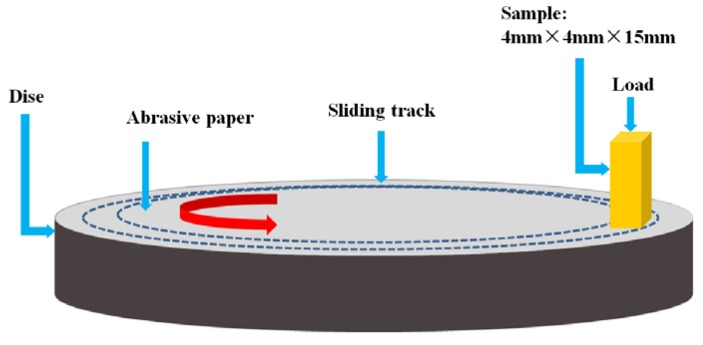
Schematic image of the pin-on-disc wear test.

The wear test was performed on a static-load pin wear tester (ML-100, Beilun, Zhangjiakou, China) with a sliding distance of 24.78 m at room temperature. The wear test schematic is shown in Figure 2. The cermets were machined into block samples with dimensions of 4 × 4 × 15 mm^3^, and the 4 × 4 mm^2^ faces were used as the wear surfaces. Al_2_O_3_ abrasive papers with abrasive particle sizes of ~10 μm, ~20 μm, and ~45 μm were used. The applied normal loads were 15, 25, and 35 N and the sliding distance was kept constant at 24.78 m. The weight loss was measured before and after the wear test using an electronic balance with a resolution of 0.1 mg, and the density of the cermets was measured by Archimedes’ water-immersion method. The worn surfaces were examined by SEM. The abrasive surface roughness of composites in this work was tested by the confocal laser scanning microscope (CLSM, Model Olympus LEXT OLS3000, Olympus, Tokyo, Japan), and surface roughness (Rt) represents the height different between the highest point and lowest point on the worn surface after the wear test. 

## 3. Results and Discussion

**Figure 3:**
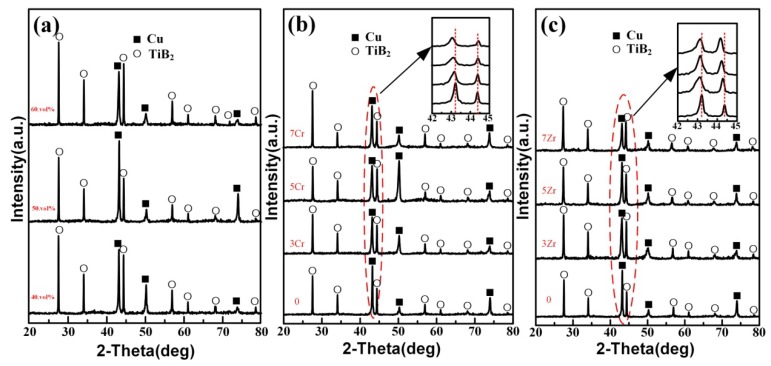
X-ray diffraction (XRD) patterns of the TiB_2_/Cu cermets: (**a**) 40-60 vol % TiB_2_/Cu cermets; (**b**) 50 vol % TiB_2_/Cu cermets with different Cr contents (Cr content from bottom to top is 0, 3, 5, and 7 wt %, respectively); (**c**) 50 vol % TiB_2_/Cu cermets with different Zr contents (Zr content from bottom to top is 0, 3, 5, and 7 wt %, respectively).

Figure 3 shows the XRD patterns of TiB_2_/Cu cermets with different TiB_2_, Zr, and Cr contents, respectively. Figure 3a shows the XRD patterns of 40, 50, and 60 vol % TiB_2_/Cu cermets. It can be seen that all of the samples were pure and contained only two phases, Cu and TiB_2_, without a brittle intermetallic phase, indicating that pure cermets could be successfully fabricated by CS and hot press consolidation of Cu-Ti-B systems. Figure 3b shows the XRD patterns for the 50 vol % TiB_2_/Cu cermets with 0, 3, 5, and 7 wt % Cr addition. The insert figure in Figure 3b shows that the peak position of the Cu (111) plane becomes wider and shifts gradually to the left, with the increase in Cr content. This shift occurs because the atomic radius of Cr (r = 0.185 nm) is larger than that of Cu (r = 0.157 nm), so that when the added Cr is dissolved into the Cu matrix, the corresponding diffraction angle of Cu decreases. Figure 3c shows the XRD patterns for the 50 vol % TiB_2_/Cu cermets with 0, 3, 5, and 7 wt % Zr addition. Unlike in the case of Cr addition, with the increase in the Zr content, the peaks of the Cu (111) plane and TiB_2_ (101) plane shifted to the left simultaneously because the atomic radius of Zr (r = 0.216 nm) is larger than that of both Cu (r = 0.157 nm) and Ti (r = 0.200 nm). Both peaks shifted because the Zr can be solid-dissolved not only into the Cu lattice, but also into the TiB_2_ lattice, so that Zr was substituted into the Ti positions, causing lattice distortion. The addition of Zr was also beneficial to the interfacial bonding between Cu and the reinforcement phase. Additions of Zr, modify the bulk activities in the alloy and adsorb at the solid–liquid interface, increasing its “metalliticity” and so promoting wetting, and this has recently been investigated by Muolo et al. [50,51,52]. 

**Figure 4:**
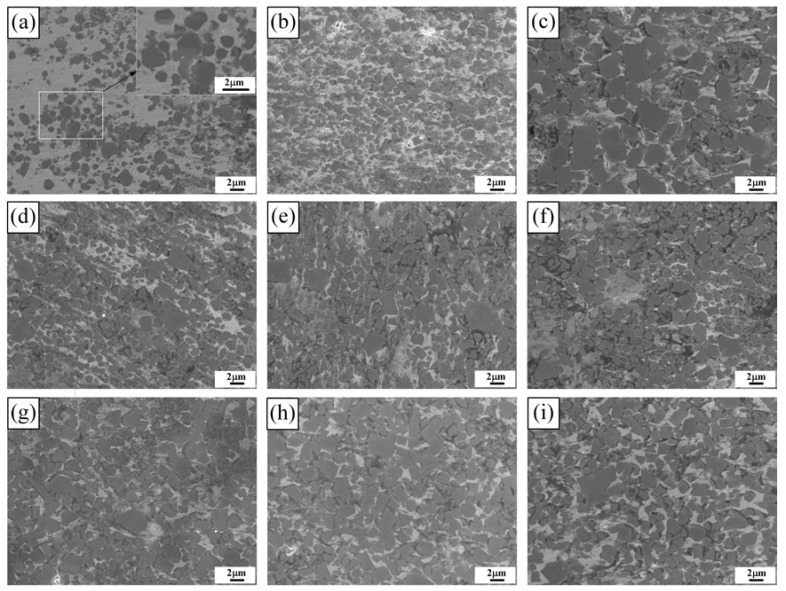
Scanning electron microscopy (SEM) images of the TiB_2_/Cu cermets with different Cr and Zr contents, (**a**–**c**) TiB_2_/Cu cermets with 40, 50, and 60 vol % TiB_2_, respectively; (**d**–**f**) 50 vol % TiB_2_/Cu cermets with 3, 5, and 7 wt % Cr, respectively; (**g**–**i**) 50 vol % TiB_2_/Cu cermets with 3, 5 and 7, wt % Zr, respectively.

**Figure 5:**
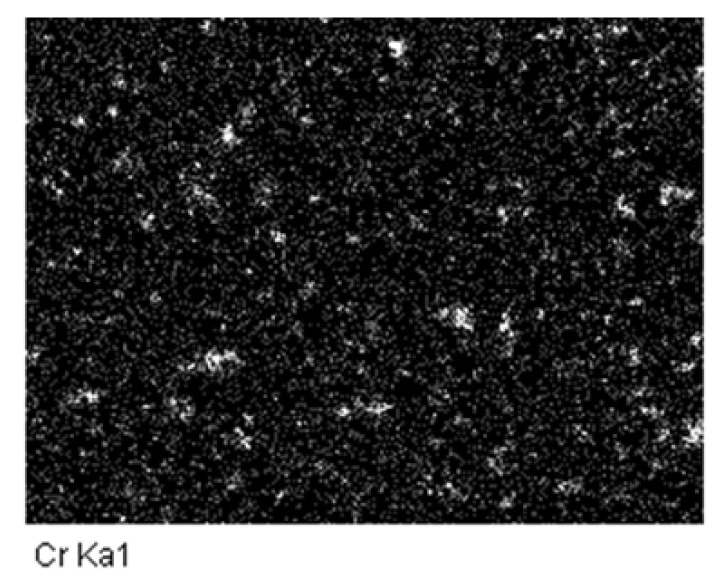
EDS analysis, element mappings of Cr in the 50 vol % TiB_2_/Cu cermets with 7 wt % Cr.

Figure 4 shows the SEM images of the 40, 50, and 60 vol % TiB_2_/Cu cermets (a–c) with different Cr (d–f) and Zr contents (g–i). As shown in Figure 4a–c, the hexagonal columnar TiB_2_ particles are uniformly distributed in the Cu matrix, the interfacial contact between the reinforcement and the copper matrix was good, and the size of the ceramic particles gradually increased with the increase in TiB_2_ content. Figure 4d–f show SEM images of the 50 vol % TiB_2_/Cu cermets with 3, 5, and 7 wt % Cr addition. With the increase in Cr content, the size of the ceramic particles does not change greatly. However, when adding 7 wt % Cr to the Cu-Ti-B system, segregation of locally aggregated Cr occurs at the interface between TiB_2_ and copper. A large number of dark black areas appear in the Figure 4f. By the energy dispersive spectrometer (EDS) analysis of the 50 vol % TiB_2_/Cu cermets with 7 wt % Cr (Figure 5), the segregation of Cr elements corresponded to the black area in the Figure 4f. This could be expected to influence the mechanical properties and wear properties of the cermets, which will be confirmed in the following material properties analysis. Figure 4g–i show the SEM images of the 50 vol % TiB_2_/Cu cermets with 3, 5, and 7 wt % Zr addition. After the addition of Zr, the ceramic particles were uniformly distributed in the matrix, and were well bonded to the Cu. A small amount of segregation was also observed when the Zr content ≥ 5 wt %, but the degree of segregation was considerably smaller than that observed when adding Cr at the same weight percentage.

**Figure 6:**
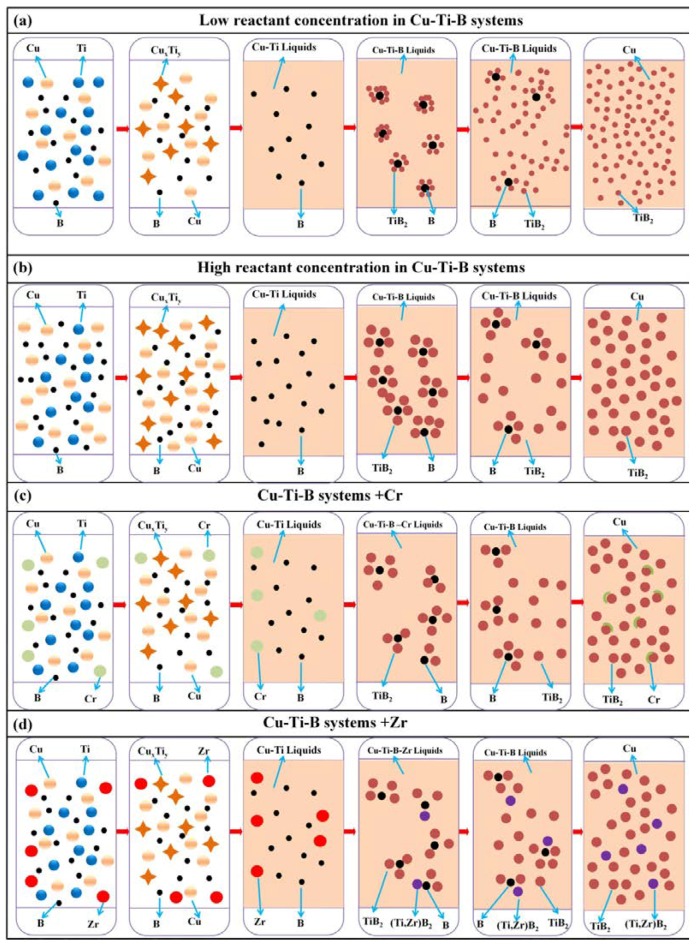
Reaction mechanism diagram of Cu-Ti-B systems, (**a**,**b**) are reaction mechanisms of Cu-Ti-B systems with high and low reactant contents, respectively; (**c**,**d**) are reaction mechanisms of Cu-Ti-B systems with Cr and Zr, respectively.

Figure 6 is a diagram of the self-propagating high-temperature synthesis (SHS) reaction mechanism for the Cu-Ti-B systems. Figure 6a,b are the reaction mechanisms of Cu-Ti-B systems with low and high reactant contents, respectively. In the first step, Cu and Ti generate a Cu*_x_*Ti*_y_* intermetallic compound through a diffusion reaction. As the temperature increases, Cu*_x_*Ti*_y_* melts to form a Cu-Ti liquid phase. Then, B diffuses into the liquid phase to form the Cu-Ti-B ternary liquid phase, while the TiB_2_ ceramic particles precipitate out of the liquid phase and grow. The size of the ceramic particles gradually increases as the TiB_2_ content increases (as shown in Figure 6a,b), for two reasons. First, with the decrease in Cu content, the amount of TiB_2_ particles synthesized by the reaction gradually increases, accompanied by a greater release of heat, resulting in an increase in the combustion temperature of the system, and the size of the ceramic particles. Second, with the decrease in Cu content, the thermal conductivity of the TiB_2_/Cu cermets decreases, which slows down the cooling of the reaction product. Therefore, the material remains at a high temperature for a longer period, resulting in more energy and time for TiB_2_ particle growth, which allows the ceramic particles to grow larger. Figure 6c,d are the reaction mechanisms of Cu-Ti-B systems with Cr and Zr additives, respectively. When adding Cr into the Cu-Ti-B systems, the quaternary liquid phase of Cu-Ti-B-Cr is formed in the reaction. Cr does not participate in the reaction, but it has an inhibitory effect on the synthesis process by diluting the reactant. Finally, a fraction of the Cr atoms enter the Cu matrix to form a solid solution in Cu, while the remaining Cr atoms are segregated around the interface between the TiB_2_ ceramic particles and Cu. The greater the amount of Cr that is added, the more serious the segregation becomes. In contrast, when Zr is added to the Cu-Ti-B systems, Zr actively participates in the SHS reaction. Eventually, Zr not only dissolves into the Cu matrix, but it also substitutes at some of the lattice positions of Ti in TiB_2_ to form the (Ti, Zr)B_2_ solid solution. Therefore, with the addition of Zr in the TiB_2_/Cu cermets, the solid solution is reinforced in the ceramic and copper phases, and the wettability between TiB_2_ and copper is also improved.

**Figure 7:**
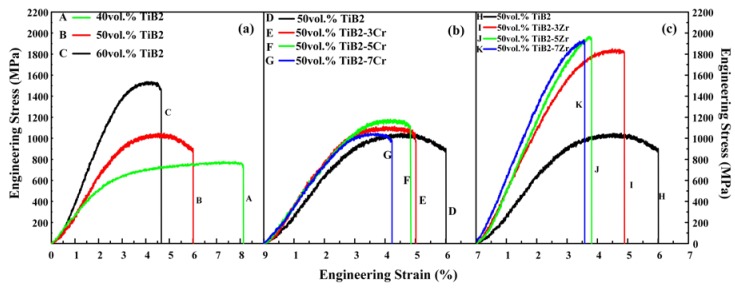
Compression engineering stress-strain curves of the TiB_2_/Cu cermets, (**a**) TiB_2_/Cu cermets with different TiB_2_ contents; (**b**) 50 vol % TiB_2_/Cu cermets with different Cr content; (**c**) 50 vol % TiB_2_/Cu cermets with different Zr content.

**Figure 8:**
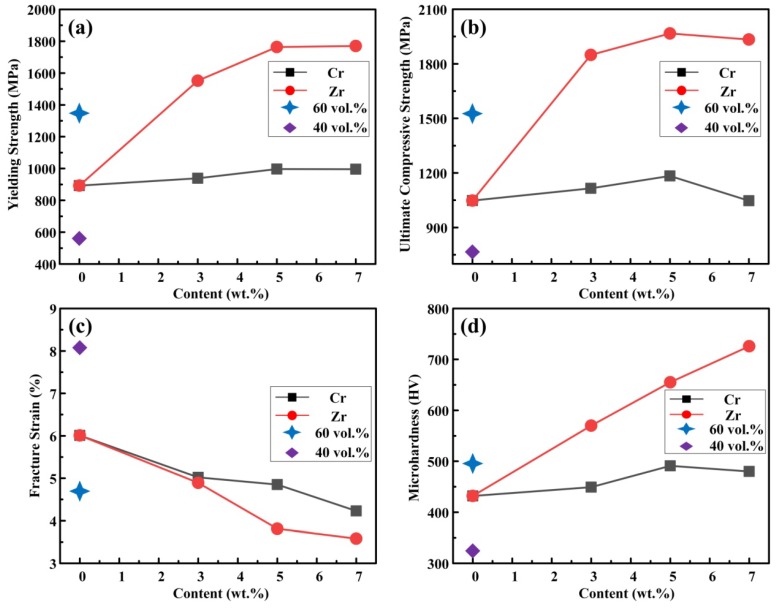
Variation in (**a**) yield strength (σ_0.2_), (**b**) ultimate compressive strength (σ*_UCS_*), (**c**) fracture strain (ε*_f_*), and (**d**) microhardness (Hv) of TiB_2_/Cu cermets with different Cr/Zr contents.

Figure 7 shows the compressive engineering stress-strain curves of TiB_2_/Cu cermets with different TiB_2_, Cr, and Zr contents prepared by CS and hot pressing in Cu-Ti-B systems. The mechanical properties and microhardness of the cermets are summarized in Table 1. The yield strength (σ_0.2_), ultimate compressive strength (σ*_UCS_*), and microhardness (Hv) increase with increasing TiB_2_ content from 40 to 60 wt %, while the fracture strain (ε*_f_*) decreases. These trends indicate that the strength of the cermets is determined by the strength of the ceramic particles and of the bonding between the ceramic and the Cu. The TiB_2_ ceramic particles have a very high strength. Therefore, as their content increases, the cermets gradually gain compressive strength. As shown in Figure 7a with the TiB_2_ content increases, the cermets gradually gain compressive strength, the compression strength is getting higher and higher, and the plasticity gradually decreases. These trends indicate that the strength of the cermets are determined by the strength and number of the ceramic particles, and the decrease in the metal binder leads to a decrease in plasticity. As shown in Figure 7b,c, the 50 vol % TiB_2_/Cu cermets obtain a significant improvement in mechanical properties and microhardness with the addition of Cr and Zr. With an increase in Cr content from 0 to 7 wt %, the σ_0.2_ and Hv increase from 893 MPa and 432 Hv to 996 MPa and 480 Hv, respectively, while the ε*_f_* decreases from 6.01% to 4.23%. With an increase in Zr content from 0 to 7 wt %, the σ_0.2_, σ*_UCS_*, and Hv increase from 893 MPa, 1048 MPa, and 432 Hv to 1770 MPa, 1934 MPa, and 726 Hv, respectively, while the ε*_f_* decreases from 6.01% to 3.58%. Figure 8 shows the variation in (a) yield strength, (b) ultimate compressive strength, (c) fracture strain, and (d) microhardness of TiB_2_/Cu cermets with different Cr/Zr contents. The addition of Zr is more effective than the addition of Cr for improving the yield strength, ultimate compressive strength, and microhardness of the TiB_2_/Cu cermets. The solid solution of Cr in Cu distorts the Cu crystal lattice, so that the stress field becomes larger, and the dislocation movement is hindered. Therefore, as the Cr content increases, both the σ_0.2_ and Hv increase. When the Cr content exceeds 5 wt %, continued addition of Cr increases the brittleness of the material, resulting in a decrease in σ*_UCS_* and Hv. When Zr is added, the Zr atoms are not only dissolved in the Cu matrix, which distorts the Cu crystal lattice, increases the stress field, and further impedes the dislocation movement, but they also form a solid solution in the TiB_2_ crystal lattice, improving the wettability of the Cu matrix and ceramic particles, and thereby increasing the bonding strength between those two phases. Therefore, the addition of Zr affords a significant improvement in the compressive strength and microhardness of the TiB_2_/Cu cermets. However, excessive addition of Zr and Cr alloying elements would lead to segregation of those elements in the matrix of the TiB_2_/Cu cermets. This would reduce the strengthening effect and sacrifice the plasticity.

**Figure 9:**
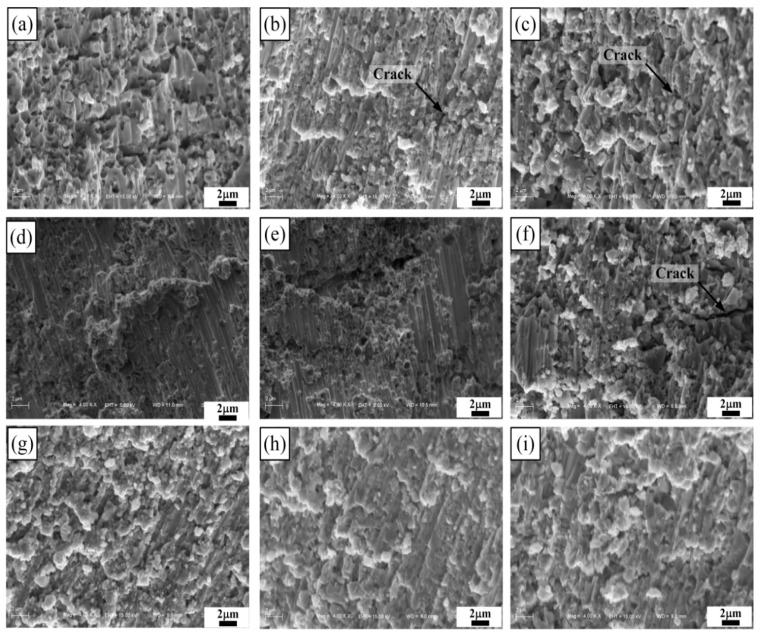
SEM images of the compression fractured surfaces for TiB_2_/Cu cermets with different Cr and Zr contents, (**a**–**c**) TiB_2_/Cu cermets with 40, 50, and 60 vol % TiB_2_, respectively; (**d**–**f**) 50 vol % TiB_2_/Cu cermets with 3, 5, and 7 wt % Cr, respectively; (**g**–**i**) 50 vol % TiB_2_/Cu cermets with 3, 5, and 7 wt % Zr, respectively.

Figure 9 shows the SEM images of the compression fractured surfaces for TiB_2_/Cu cermets with different Cr and Zr contents. Figure 9a–c shows those images for TiB_2_/Cu cermets with 40, 50, and 60 vol % TiB_2_, respectively. The ceramic particles were well coated by the Cu, and the interfacial contact between the TiB_2_ particles and the Cu was evidently close. As the ceramic content increased, the compressive strength of the cermets gradually increased. However, the increase in the content of ceramics was accompanied by a decrease in the content of Cu, which increased the brittleness of the cermets and decreased the strain. As shown in Figure 9a, when the content of TiB_2_ is 40 vol %, many dimples appear in the fracture, indicating that the cermet is relatively tough. The toughness of the cermets gradually deteriorates with an increase in the ceramic content. When the TiB_2_ content reaches 60 vol % as shown in Figure 9c, the material exhibits a brittle fracture morphology. Figure 9d–f show SEM images of the compression fractured surfaces for 50 vol % TiB_2_/Cu cermets with 3, 5, and 7 wt % Cr, respectively. Fewer dimples are observed at the fractures, and the materials are more brittle than those with no addition of Cr. When the Cr content reaches 7 wt %, as shown in Figure 9d, cracks appear in the fracture, indicating that the brittleness of the cermets has increased, and the microhardness and compression performance have deteriorated. Figure 9g–i shows SEM images of the compression fractured surfaces for 50 vol % TiB_2_/Cu cermets with 3, 5, and 7 wt % Zr, respectively. Zr can not only dissolve in the Cu matrix, but can also form a solid solution in the TiB_2_ lattice, which improves the wettability of Cu and ceramic particles, thereby improving the bonding strength between Cu and the reinforcing phase. This is beneficial for interfacial bonding and the prevention of crack propagation. With an increase in Zr content, the brittleness of the cermets increases, and the cermets eventually fracture due to lattice distortion caused by the addition of Zr and segregation of Zr atoms in the Cu matrix.

**Figure 10:**
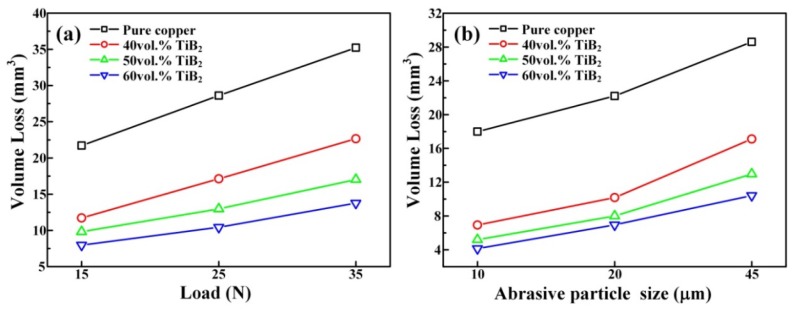
Variation in volume loss of the pure copper and the TiB_2_/Cu cermets with different loads and different abrasives, (**a**) different loads using 45 μm abrasive; (**b**) different abrasives using a load of 25 N.

**Figure 11:**
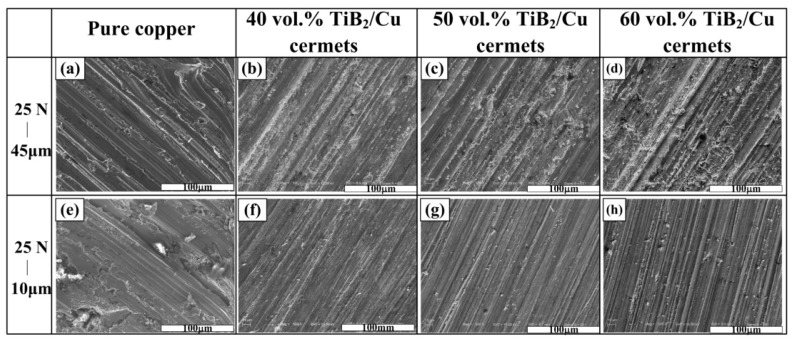
Worn surfaces of the pure copper and cermets under 25 N load with different abrasive grain sizes (μm): pure copper with (**a**) 45 μm and (**e**) 10 μm; 40 vol % TiB_2_/Cu cermets with (**b**) 45 μm and (**f**) 10 μm; 50 vol % TiB_2_/Cu cermets with (**c**) 45 μm and (**g**) 10 μm; 60 vol % TiB_2_/Cu cermets with (**d**) 45 μm and (**b**) 10 μm.

Figure 10 shows the variation in volume loss of the pure copper and TiB_2_/Cu cermets under different loads (15–35 N) or different abrasive particle sizes (10–45 μm). Figure 11 shows the worn surface of pure copper and TiB_2_/Cu cermets at 25 N load and different abrasive grain sizes. As shown in Figure 10a, the volume wear of pure copper and TiB_2_/Cu cermets increased as the load increased. Notably, as the load is increased, the volume loss of the TiB_2_/Cu cermets increased less than that of pure Cu, i.e., the cermets were less affected by the load change than was pure Cu. The volume fraction of ceramic particles increased and the volume loss decreased markedly. As shown in Figure 11a–d, the wear surfaces of the TiB_2_/Cu cermets were relatively smooth and no deep furrows were visible, in contrast to the pure Cu. This smoothness reflects the fact that with increasing volume fraction of the ceramic particles, the materials became considerably harder. The high hardness of the TiB_2_ ceramic particles in the cermets strongly impeded the ability of the Al_2_O_3_ abrasive particles to cut into the matrix. Therefore, the depth to which the abrasive particles are pressed into the surface of the material decreases, and the microscopic cutting action of the abrasive particles during sliding was also inhibited. As the load increased, the abrasive particles were pressed deeper into the surface, and their microscopic cutting action during sliding was enhanced.

Figure 10b shows the variation in volume loss of the pure copper and the TiB_2_/Cu cermets with different abrasive particle sizes using a load of 25 N. The volume loss of both pure copper and the cermets increased with an increase in abrasive particle size, but the wear resistance of the TiB_2_/Cu cermets was better than that of pure copper. As can be seen by comparing Figure 11a with e, and Figure 11b with f, with the increase in grain size of the Al_2_O_3_ abrasives, the wear surface of the TiB_2_/Cu cermets became rougher, and the width and depth of the furrows increased significantly. As the abrasive particle size increased, the material underwent more severe plastic deformation and cutting, resulting in increased volume loss of the material. The high hardness of the TiB_2_ ceramic particles in the cermets strongly impeded the cutting of the Al_2_O_3_ abrasive particles into the matrix when the abrasive grain size increased. Therefore, the plastic deformation of the cermets is reduced, and the micro-cutting effect of the Al_2_O_3_ abrasive grains was weakened, so that the cermets underwent less volume wear than pure copper.

**Figure 12:**
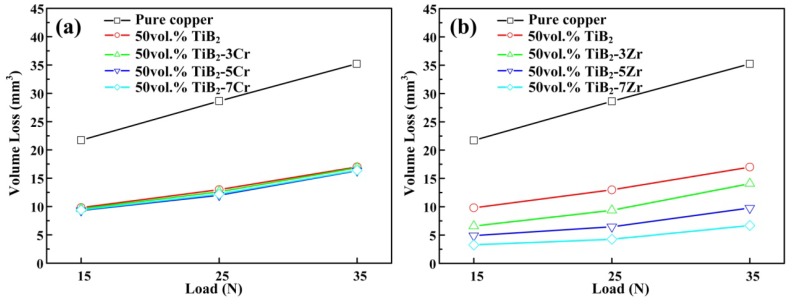
Variation in volume loss of the 50 vol % TiB_2_/Cu cermets with different Cr and Zr contents under different applied loads. (**a**) TiB_2_/Cu cermets with different Cr contents, (**b**) TiB_2_/Cu cermets with different Zr contents.

**Figure 13:**
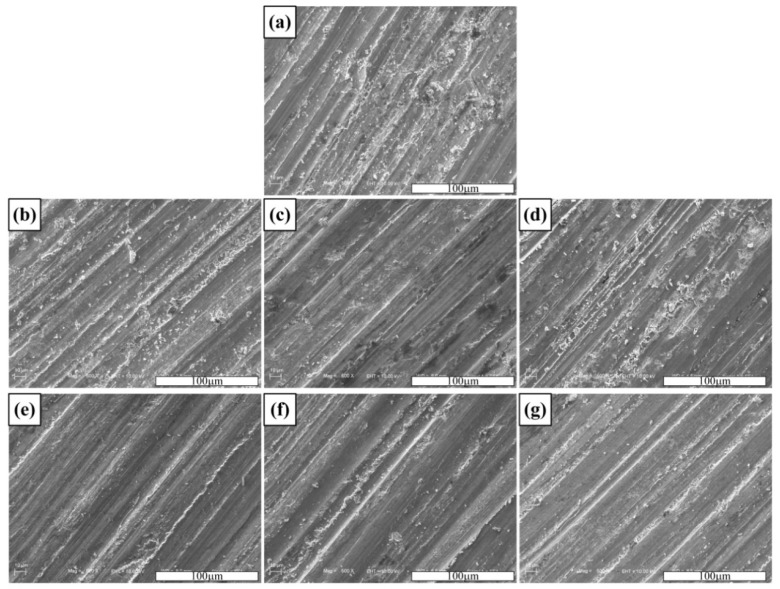
Worn surface of the 50 vol % TiB_2_/Cu cermets with different Cr and Zr contents under a 25 N applied load and 45 μm abrasive particles. (**a**) 50 vol % TiB_2_/Cu cermets, (**b**–**d**) 50 vol % TiB_2_/Cu cermets with 3, 5, and 7 wt % Cr, respectively, (**e**–**g**) 50 vol % TiB_2_/Cu cermets with 3, 5, and 7 wt % Zr, respectively.

Figure 12a,b show the variation in volume loss with applied load for the 50 vol % TiB_2_/Cu cermets with different Cr and Zr contents, respectively. Figure 13 shows the worn surface of the 50 vol % TiB_2_/Cu cermets with different Cr and Zr contents under a 25 N applied load and 45 μm abrasive particles. As shown in Figure 12, the addition of alloying elements Cr and Zr increased the wear resistance of the cermets. As shown in Figure 13, the cermets alloyed with Zr had smoother wear surfaces than those alloyed with Cr. The 50 vol % TiB_2_/Cu cermets obtained a smaller mean surface roughness of the worn surfaces with the addition of Cr and Zr. With an increase in Cr contents from 0 to 7 wt %, the values of the mean surface roughness of the worn surfaces were 3.487 μm, 2.985 μm, 2.648 μm, and 2.328 μm, respectively. With an increase in Zr contents from 0 to 7 wt %, the values of the mean surface roughness of the worn surfaces were 3.487 μm, 1.357 μm, 1.109 μm, and 0.932 μm, respectively. The cermets alloyed with Zr had smoother wear surfaces and smaller mean surface roughness of the worn surfaces than those alloyed with Cr, which indicates that the former had better wear resistance. There are two main reasons for this difference. First, in contrast to the cermets alloyed with Cr, the alloyed Zr content was not only dissolved in the Cu matrix, but also, because Zr was in the same family of elements as Ti, it could be dissolved in TiB_2_ to form a (Ti, Zr)B_2_ solid solution. This further enhances the solid solution strengthening effect of Zr on the cermets. Second, the addition of Zr significantly improved the wettability between the Cu and the reinforcing phase, thereby increasing the interfacial bonding strength between those phases, and this effect became more pronounced as the Zr content increased. 

**Figure 14:**
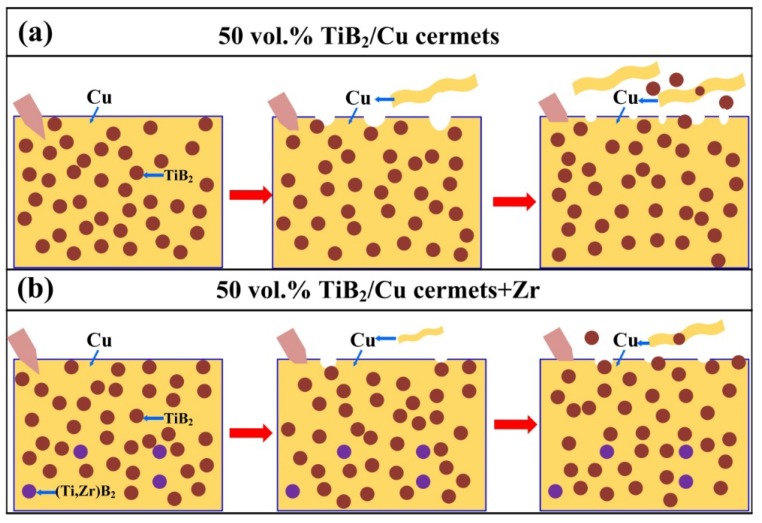
Abrasive wear mechanism for (**a**) 50 vol % TiB_2_/Cu cermets and (**b**) 50 vol % TiB_2_/Cu cermets with Zr addition.

Figure 14 shows the abrasive wear mechanism for (a) 50 vol % TiB_2_/Cu cermets and (b) the 50 vol % TiB_2_/Cu cermets with Zr addition. The wear mechanism in the unalloyed TiB_2_/Cu cermets (Figure 14a) is as follows. First, when the cermet comes into contact with the sandpaper, the hard Al_2_O_3_ abrasive penetrates into the relatively soft Cu, causing a large amount of Cu to peel off from the surface of the cermet. At the same time, the hard TiB_2_ particles impact somewhat on the Al_2_O_3_ abrasive particles, which passivates the latter to some extent. After the TiB_2_ particles are exposed on the surface of the cermets, they strongly hinder the micro-cutting of the Al_2_O_3_ abrasive particles into the cermets. However, once the exposed TiB_2_ particles are completely removed from the surface of the cermets, the Al_2_O_3_ particles can once again cut into the Cu and TiB_2_ particles. 

The wear strengthening mechanism of the TiB_2_/Cu cermets with the addition of Zr (Figure 14b) was as follows. First, the solid solution of Zr in Cu acted to strengthen the material: as can be seen from the figure, the cermets alloyed with Zr were pierced to a shallower depth by the abrasive grains than the cermet without the addition of Zr. As well as strengthening the ceramic particles, the addition of Zr also had a more significant passivation effect on the abrasive particles. Finally, the interface between the reinforcement phase and the Cu was strengthened. The addition of Zr prevented the strengthened ceramic particles from being easily peeled off the matrix, as shown in Figure 14b. Therefore, the addition of Zr greatly improved the wear resistance of the cermets. 

## 4. Conclusions

In situ micro-TiB_2_/Cu cermets with a different TiB_2_ content (40, 50, and 60 vol %) were successfully fabricated by combustion synthesis and hot press consolidation in Cu-Ti-B-(Cr/Zr) systems. The in situ TiB_2_ particles were uniformly distributed in the matrix. The size of the ceramic particles gradually increased as the TiB_2_ content increased. With decreasing Cu content, the synthesis reaction released greater amounts of heat, resulting in an increase in the combustion temperature of the system. Therefore, the materials remained at high temperature for a longer period, providing more energy and time for TiB_2_ particle growth. When Cr and Zr were added, at the same weight percentage, the added Zr atoms showed significantly less segregation than that of Cr. After the addition of Zr, the ceramic particles were uniformly distributed in the matrix, and well bonded to the Cu. 

As the content of the TiB_2_ ceramic particles increased, the compressive strength of the cermets also gradually increased. The TiB_2_/Cu cermets obtained a significant improvement in mechanical properties and microhardness with the addition of the Cr and Zr. The addition of Zr was more effective than the addition of Cr for improving the yield strength, ultimate compressive strength, and microhardness of the TiB_2_/Cu cermets. When the Zr content was 5 wt %, the yield strength, ultimate compressive strength, and microhardness of the cermets reached 1764 MPa, 1967 MPa, and 655 HV, respectively, amounting to increases of 97%, 87%, and 52% with respect to the unalloyed cermet. The Zr content was not only dissolved in the Cu matrix, which distorted the Cu crystal lattice, increased the stress field, and further impeded the dislocation movement, but also formed a solid solution in the TiB_2_ crystal lattice, improving the wettability of the Cu and ceramic particles, thereby increasing the bonding strength between the Cu and the reinforcement phase.

The volume wear of both the pure copper and TiB_2_/Cu cermets increased with increases in load and abrasive particle size. However, with an increase in the volume fraction of ceramic particles and the addition of Zr, the volume loss was significantly reduced. The addition of Zr greatly improved the wear resistance of the cermets, mainly due to the strengthening effect of the alloyed Zr, and the formation of a solid solution of Zr in Cu, and TiB_2_ to strengthen the interfacial bonding. 

## Figures and Tables

**Table 1:** Room-temperature compression properties and microhardness of TiB_2_/Cu cermets with different TiB_2_, Cr, and Zr contents.

Samples	σ_0.2_ (MPa)	σ*_UCS_* (MPa)	ε*_f_* (%)	Hv
40 vol % TiB_2_	$579_{-12}^{+15}$	$778_{-14}^{+19}$	$8.14_{-0.2}^{+0.5}$	321 ± 8
50 vol % TiB_2_	$893_{-16}^{+20}$	$1048_{-20}^{+25}$	$6.01_{-0.1}^{+0.3}$	432 ± 10
60 vol % TiB_2_	$1385_{-18}^{+25}$	$1543_{-13}^{+21}$	$4.66_{-0.3}^{+0.2}$	493 ± 9
50 vol % TiB_2_+3Cr	$939_{-21}^{+25}$	$1115_{-23}^{+30}$	$5.02_{-0.2}^{+0.1}$	449 ± 9
50 vol % TiB_2_+5Cr	$997_{-26}^{+30}$	$1183_{-24}^{+33}$	$4.85_{-0.4}^{+0.2}$	491 ± 8
50 vol % TiB_2_+7Cr	$996_{-12}^{+21}$	$1047_{-18}^{+26}$	$4.23_{-0.4}^{+0.3}$	480 ± 10
50 vol % TiB_2_+3Zr	$1552_{-24}^{+35}$	$1849_{-28}^{+40}$	$4.89_{-0.3}^{+0.1}$	570 ± 12
50 vol % TiB_2_+5Zr	$1764_{-51}^{+44}$	$1967_{-23}^{+35}$	$3.81_{-0.3}^{+0.4}$	655 ± 11
50 vol % TiB_2_+7Zr	$1770_{-29}^{+38}$	$1934_{-24}^{+31}$	$3.58_{-0.1}^{+0.2}$	726 ± 9
